# Distribution of regional lymph lode metastasis in unilateral nasopharyngeal carcinoma and the suggestions for selective prophylactic irradiation with intensity‐modulated radiotherapy

**DOI:** 10.1002/cam4.6614

**Published:** 2023-10-25

**Authors:** Feifei Lin, Zichen Qiu, Dehuan Xie, Xiong Zhou, Lei Wang, Zheng Wu, Wanqin Cheng, Shaowen Lyu, Yong Su, Yalan Tao

**Affiliations:** ^1^ Department of Radiation Oncology Sun Yat‐sen University Cancer Center, State Key Laboratory of Oncology in South China, Collaborative Innovation Center for Cancer Medicine, Guangdong Key Laboratory of Nasopharyngeal Carcinoma Diagnosis and Therapy Guangzhou P.R. China; ^2^ Department of Radiation Oncology Clinical Oncology School of Fujian Medical University, Fujian Cancer Hospital Fuzhou China; ^3^ Department of Radiotherapy Guangdong Provincial People's Hospital, Guangdong Academy of Medical Sciences Guangdong Guangzhou P.R. China; ^4^ Department of Radiation Oncology The Third Affiliated Hospital of Kunming Medical University (Yunnan Cancer Hospital, Yunnan Cancer Center) Kunming P.R. China; ^5^ Department of Radiation Oncology, Hunan Cancer Hospital and The Affiliated Cancer Hospital 14 of Xiangya School of Medicine Central South University Changsha P.R. China; ^6^ Department of Radiation Oncology Shunde Hospital of Southern Medical University Foshan P. R. China; ^7^ Department of Radiation Oncology (MAASTRO), GROW School for Oncology and Developmental Biology Maastricht University Medical Center Maastricht The Netherlands

**Keywords:** contralateral lymph node, intensity‐modulated radiotherapy, regional lymph node metastasis, selective prophylactic irradiation, unilateral nasopharyngeal carcinoma

## Abstract

**Purpose:**

To address the regional lymph node (RLN) distribution and the long‐term efficacy in unilateral nasopharyngeal carcinoma (NPC), providing elective irradiation for RLN with intensity‐modulated radiotherapy (IMRT).

**Methods:**

The involvement of clinical data of 136 patients with unilateral NPC, who underwent IMRT from November 2003 to December 2020 were analyzed retrospectively. The therapeutic effect and failure pattern of RLN metastasis were evaluated.

**Results:**

Of 57.1% patients have bilateral RLN metastasis. The rate of contralateral RLNs metastasis is lower than that of ipsilateral RLNs. Contralateral RLN metastasis mainly occurs in level VIIa (39.0%) and II (38.2%). While level IVa is only 0.7%, and none of RLN metastasis was found in level IVb and Va. The median follow‐up was 70 months, and the 3‐, 5‐and 10‐year regional recurrence‐free survival (RRFS) were 94.1%, 93.1%, and 93.1%, respectively.

**Conclusion:**

Routine prophylactic irradiation may not include contralateral lower neck LN and level Va for N0‐1 unilateral NPC.

## 1 BACKGROUND

Intensity modulated radiotherapy (IMRT) is the primary treatment for nasopharyngeal carcinoma (NPC).^1^ More than 80% of NPC patients have regional lymph node (RLN) metastasis at the time of diagnosis, and approximately 50% have bilateral RLN metastasis.^2^, ^3^ Therefore, most international research centers^4^, ^5^, ^6^ recommend that prophylactic whole‐neck irradiation should be performed for any stage. However, there are few studies on how to carry out selective prophylactic irradiation of bilateral neck in unilateral NPC, which was confined to the one side of the nasopharynx by fiberoptic endoscopy and magnetic resonance imaging (MRI). Due to the particularity of unilateral NPC, the distribution of RLN metastasis may be different. Therefore, an individualized principle of clinical target delineation is worth exploring for unilateral NPC. In a previous study,^7^ our team discussed the method of clinical target volume delineation of the primary focus of unilateral NPC with IMRT. On this basis, we further analyze the distribution of RLN metastasis and the treatment failure pattern, in order to provide a reference for the delineation of RLN targets in unilateral NPC.

## 2 MATERIALS AND METHODS

### 2.1 Patient characteristics

From November 2003 to December 2020, a total of 647 biopsy‐proven NPC patients treated with IMRT in our research group, of which 136 with primary unilateral NPC were recruited. The inclusion criteria were: (1) pathologically and initially confirmed unilateral NPC; (2) >18 years old; (3) received the IMRT; (4) the performance status (PS) of 0 or 1; (5) without distant metastasis; (6) informed consent form related to treatment signed. Exclusion criteria: (1) metachronous or synchronous malignant tumors; (2) pregnancy or lactation; (3) mental disorders. Retrospective ethical approval was obtained from the institutional ethics committee of the Sun Yat‐sen University Cancer Center, with the approval number YB2019‐169‐01.

### 2.2 Diagnostic criteria

All patients underwent MRI or electronic nasopharyngoscope (ENS) before treatment. Unilateral NPC was defined as the primary tumor that was confined to one side and did not exceed the midline of the nasopharynx.

All patients were examined by electronic nasopharyngoscopy and MRI. The criteria for RLN metastasis^8^ were as follows: (1) a lymph node (LN) ≥ 10 mm; (2) central necrosis or marginal circular enhancement or extracapsular invasion of LNs of any size; (3) a cluster of three LNs in the same region, and the minimum diameter of one at least ≥8 mm; (4) the shortest axial diameter of retropharyngeal LNs ≥5 mm. The standard of cervical LN zoning was defined by Grégoire et al.^9^

### 2.3 MRI scan

All patients received the head and neck MR examination with a scan range from the suprasellar cistern to the inferior margin of the sternal end of the clavicle. Patients underwent MRI by a 1.5‐T MRI unit ([Signa Excite, Signa HDx; GE]; [Symphony; Siemens]; [Achieva, PHILIPS]) or 3‐T MRI unit ([Trio Tim; SIEMENS]; [Achieva, PHILIPS]; [Discovery MR750, Discovery MR750w; GE]; [Verio; Siemens]). T1‐ and T2‐weighted fast spin echo images in the axial were obtained before injection of contrast material. Enhanced T1WI axial sequences were performed 40 seconds after intravenous Gd‐DTPA (Magnevist; Bayer Schering Pharma AG) injection at a dose of 0.1 mmol/kg. In order to obtain more accurate diagnostic results, MRI should be completed within a reasonable time frame, and efforts should be made to shorten the scan time while ensuring image quality.

### 2.4 Radiotherapy

**FIGURE 1:**
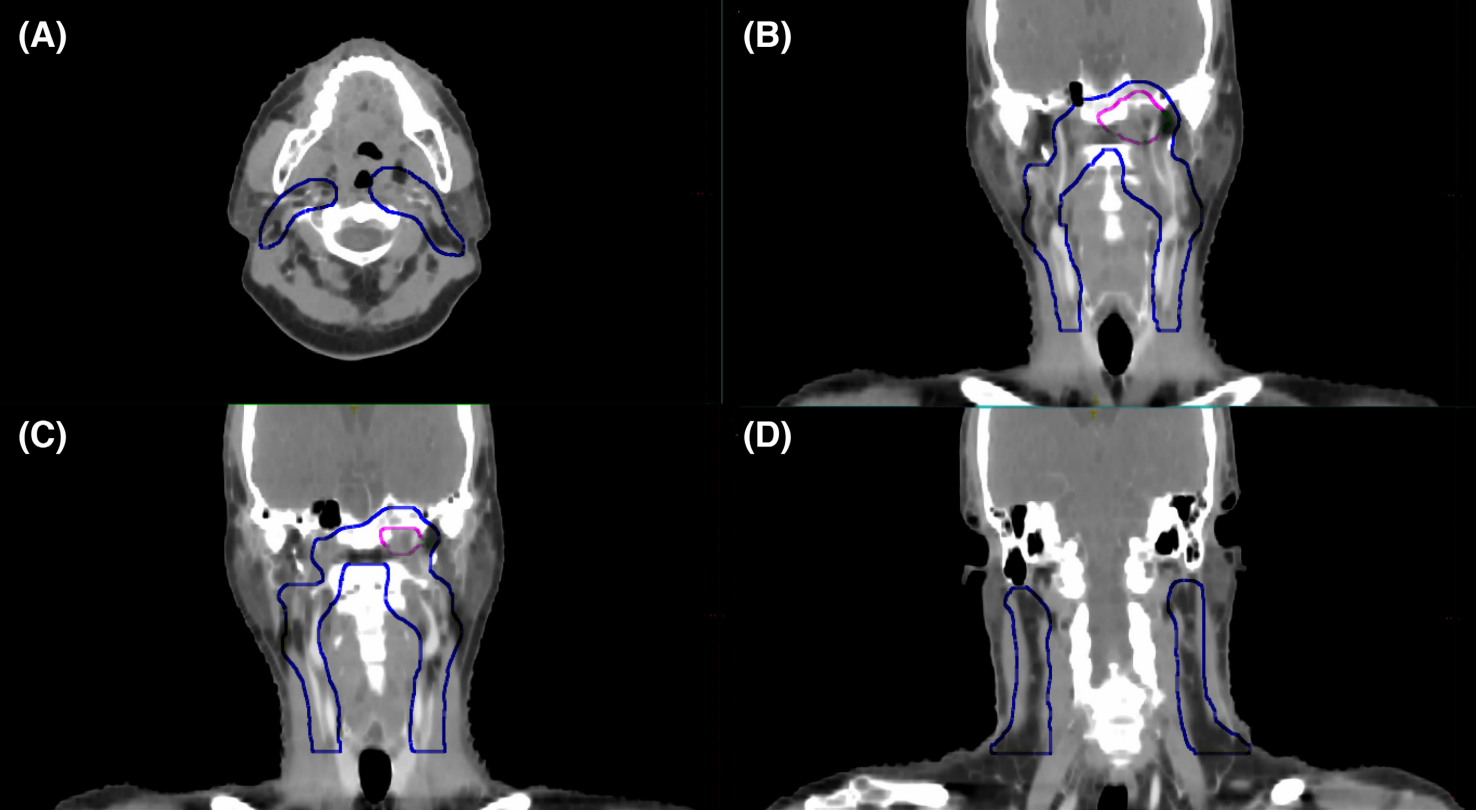
Irradiation range of CTV2 in RLN drainage area for N0 patients with unilateral NPC. The blue line area is the range of CTV2, the pink line area is the range of CTV1.

All patients were treated with IMRT which was based on the consensus guideline^1^, ^10^ and the practice in our hospital.^7^, ^11^ Target volume delineation: gross tumor volume of the metastatic lymph node (GTVnd) was defined as positive metastatic LNs detected clinically, the high‐risk CTV (high‐risk clinical target volume of LNs, CTV1) was defined as GTV + 5–10 mm. The low‐risk CTV (CTV2) was defined as having a 5–10 mm margin surrounding CTV1, including the bilateral prophylactically irradiated LN drainage areas which was based on our previous studies^7^, ^12^ (Figure 1 shows the CTV2 area of N0 patients). Planning target volume (PTV) was created by expanding 3 mm from all target volumes from the head‐to‐foot, front‐to‐back, and left‐to‐right directions around the target volumes mentioned above to compensate for geometric uncertainties and patient movements, such as PGTVnd (Planning target volume of metastatic LN), PCTV1(Planning high‐risk clinical target volume of LNs), and PCTV2 (Planning low‐risk clinical target volume).

### 2.5 Chemotherapy

Induction chemotherapy: TPF (docetaxel 60 mg/m^2^, cisplatin 60 mg/m^2^, fluorouracil 500 mg/m^2^ continuous venous perfusion for 120 h), three to four times in total. Concurrent chemotherapy: a weekly regimen containing platinum, including cisplatin (30 mg/m^2^), nedaplatin (30 mg/m^2^), four to six times in total; concurrent chemotherapy, including cisplatin weekly regimen (30 mg/m^2^), four to six times in total and cisplatin three‐week regimen (80 mg/m^2^), three to four times in total.

### 2.6 Follow‐up

All patients were followed up every 3 months during the first 3 years, every 6 months after 3–5 years, and once a year after 5 years. Recurrence was confirmed by imaging and pathological biopsy. According to methods described in the literature, failure patterns were defined and classified as “in‐field”, “marginal” and “out‐of‐field”.^13^, ^14^

### 2.7 Statistical analysis

Statistical Package for the Social Sciences (SPSS) software version 24.0 was used for all statistical analyses. To test the homogeneity of variance, the continuous variables with normal distribution and homogeneity of variance were tested using the *t*‐test, and their distribution characteristics are described by the mean and standard deviation; otherwise, nonparametric tests such as the Wilcoxon rank sum test were used. For classification variables, the chi‐squared test or Fisher's exact test was selected according to the frequency. Survival analysis was calculated using the Kaplan–Meier method, and the Log‐Rank test was used to compare the differences. The Cox proportional regression model was used for univariate and multivariate analysis, and the risk ratio (Hazard Ratio, HR) and the corresponding confidence interval (CI) were calculated. *p* < 0.05 was considered statistically significant.

## 3 RESULTS

### 3.1 Patient characteristics

The baseline characteristics of 136 unilateral NPC patients are shown in Table 1. The lesions in 47.1% of patients (64/136) were on the left side, and 52.9% (72/136) were on the right side. The median age of the patients was 47 (18–77) years. The percentages of N0, N1, N2, and N3 patients were 7.4%, 38.2%, 39.7%, and 14.7%, respectively. T1, T2, T3, and T4 accounted for 26.5%, 19.9%, 45.6%, and 8.0%, respectively.

**TABLE 1:** Clinical characteristics.

	No. of patients (%) Total = 136
Sex	
Male	97 (71.3)
Female	39 (28.7)
Median age (range)	47 (18–77)
Pathology	
Non‐keratinized carcinoma	133 (97.8)
Keratinized carcinoma	3 (2.2)
Site	
Left	64 (47.1)
Right	72 (52.9)
T stage	
T1	36 (26.5)
T2	27 (19.9)
T3	62 (45.6)
T4	11 (8.0)
N stage	
N0	10 (7.4)
N1	52 (38.2)
N2	54 (39.7)
N3	20 (14.7)
M stage	
M0	134 (98.5)
M1	2 (1.5)
Clinic stage	
I	6 (4.4)
II	25 (18.4)
III	74 (54.4)
IVA	29 (21.3)
IVB	2 (1.5)
EBER expression status	
Positive	114 (83.8)
Negative	5 (3.7)
Unknown	17 (12.5)
Treatment	
RT alone	22 (16.2)
IC + CCRT	64 (47.0)
IC + RT	50 (38.2)

Abbreviations: CCRT, Concurrent chemoradiotherapy; EBER, Epstein–Barr virus‐encoded small RNA (ribonucleic acid); IC, induction chemotherapy; RT, radiotherapy.

### 3.2 Distribution of RLNs

The total rate of RLN metastasis was 92.6% (126/136), of which the contralateral RLN metastasis rate was 60.3% (76/126), and the bilateral RLN metastasis rate was 57.9% (73/126). In each LN region, the contralateral metastasis rate was lower than that of the ipsilateral site, and the contralateral metastatic LNs mainly occurred in level VIIa (41.9%) and II (38.2%) (the distribution of RLNs are shown in Table 2, with a risk classification of ≥35%, 5%–35%, and <5%^15^). There was no skip RLN metastasis bilaterally, metastasis was rare in contralateral IVa region (0.7%), and no metastasis was observed in contralateral level IVb and V (0%). Patients with ipsilateral level V (14.0%) metastasis were all staged N2‐3, which was also accompanied by metastasis at with ipsilateral level VIIa metastasis (100%), level II metastasis (80%), level III metastasis (80%) and level IVa metastasis (46.7%).

**TABLE 2:** Distribution of metastasis RLN.

Level	Ipsilateral RLN metastasis	Contralateral RLN metastasis	Bilateral RLN metastasis
I			
Ia	–	–	1(0.7%)
Ib	4(2.9%)	2(1.5%)	2(1.5%)
II	111(81.6%)	52(38.2%)	51(37.5%)
IIa	90(66.2%)	40(29.4%)	39(28.7%)
IIb	102(75.0%)	38(27.9%)	36(26.5%)
III	58(42.6%)	11(8.1%)	11(8.1%)
IV	17(12.5%)	1(0.7%)	0
IVa	17(12.5%)	1(0.7%)	0
IVb	2(1.5%)	0	0
V	19(14.0%)	0	0
Va	15(11.0%)	0	0
Vb	10(7.4%)	0	0
Vc	2(1.5%)	0	0
VII	115(84.6%)	57(41.9%)	54(39.7%)
VIIa	115(84.6%)	57(41.9%)	54(39.7%)
VIIb	4(2.9%)	0	0

### 3.3 Correlation of metastatic RLNs between the contralateral and ipsilateral region

The correlation between contralateral RLNs and ipsilateral ones was further analyzed and the results are shown in Table 3. Univariate analysis demonstrated that contralateral level VIIa metastasis was associated with ipsilateral level VIIa (χ^2^ = 22.393, *p* = 0.003) and IIa (χ^2^ = 4.667, *p*<0.001) metastasis, contralateral level IIa metastasis was associated with ipsilateral level IIa (χ^2^ = 30.000, *p* = 0.001) and IIb (χ^2^ = 3.030, *p* = 0.036) metastasis, and contralateral level IIb was associated with ipsilateral level IIa (χ^2^ = 14.073, *p*<0.001), IIb (χ^2^ = 8.727, *p* = 0.004) and III (χ^2^ = 5.305, *p*<0.001) metastasis. Multivariate analysis showed that contralateral level VIIa metastasis was associated with ipsilateral level VIIa (χ^2^ = 28.612, *p* = 0.004) and IIa (χ^2^ = 6.139, *p* = 0.001) metastasis, contralateral level IIa metastasis was associated with ipsilateral level IIa (χ^2^ = 52.240, *p* = 0.001) and IIb (χ^2^ = 26.774, *p* = 0.042) metastasis, and contralateral level IIb was associated with ipsilateral level IIa (χ^2^ = 9.915, *p* = 0.007) and III (χ^2^ = 3.749, *p* = 0.011) metastasis.

**TABLE 3:** Univariate and multivariate analysis of different levels between ipsilateral and contralateral RLN metastasis.

Contralateral RLN	Ipsilateral RLN (Univariate analysis)											
	VIIa	VIIb	Ib	IIa	IIb	III	IVa	IVb	Va	Vb	Vc	
VIIa	0.003*	0.219	0.254	0.001*	0.318	0.136	0.156	0.414	0.157	0.258	0.833	
IIa	0.379	0.844	0.059	0.001*	0.036*	0.136	0.260	0.873	1.000	0.242	0.730	
IIb	0.071	0.188	0.921	0.000*	0.004*	0.000*	0.200	0.999	0.275	0.497	1.00	
III	0.091	0.628	0.076	0.832	0.998	0.995	0.998	1.000	0.998	0.998	0.998	

**Contralateral RLN**	**Ipsilateral RLN (Multivariate analysis)**											
	VIIa	VIIb	Ib	IIa	IIb	III	IVa	IVb	Va	Vb	Vc	
VIIa	0.004*	. 391	0.808	0.001*	0.202	0.497	0.745	1.00	0.380	0.819	1.000	
IIa	0.970	0.230	0.700	0.001*	0.042*	0.628	0.786	0.873	1.000	0.242	0.730	
IIb	0.318	0.208	0.854	0.007*	0.419	0.011*	0.906	1.000	0.229	0.780	1.000	
III	0.091	0.658	0.069	0.997	0.997	0.995	0.094	1.000	0.159	0.706	1.000	

* indicates Statistically significant difference (p value < 0.05).

### 3.4 Risk factor association with contralateral RLN metastasis

The location of the primary focus of unilateral NPC was not significantly correlated with contralateral RLN metastasis, and had no significant correlation with T stage and tumor volume (*p* > 0.05, Table 4).

**TABLE 4:** Risk factor association with contralateral RLN metastasis.

	Contralateral LN metastasis		*p*‐value	Exp(B)	95% CI
	Yes	No			
T					
T1‐2	46	8	0.069	0.434	(0.176, 1.068)
T3‐4	50	21			
Tumor volume^a^					
≤48 mL	58	17	0.603	1.578	(0.283, 8.791)
>48 mL	38	13			
Primary site^b^			0.219		
Medial site	49	16			
Lateral site	40	10	0.554	0.510	(0.055, 4.730)
Complete lateral site	10	6	0.225	0.264	(0.031, 2.268)

^a^ According to our previous study^38^, tumor volume is divided into groups by 48 mL.

^b^ Primary focus location: the medial site has exceeded half of the ipsilateral posterior parietal wall but does not reach the midline; the lateral site does not reach half of the ipsilateral posterior parietal wall; the complete lateral site is completely confined to the ipsilateral nasopharyngeal lateral wall.

### 3.5 Survival results

**FIGURE 2:**
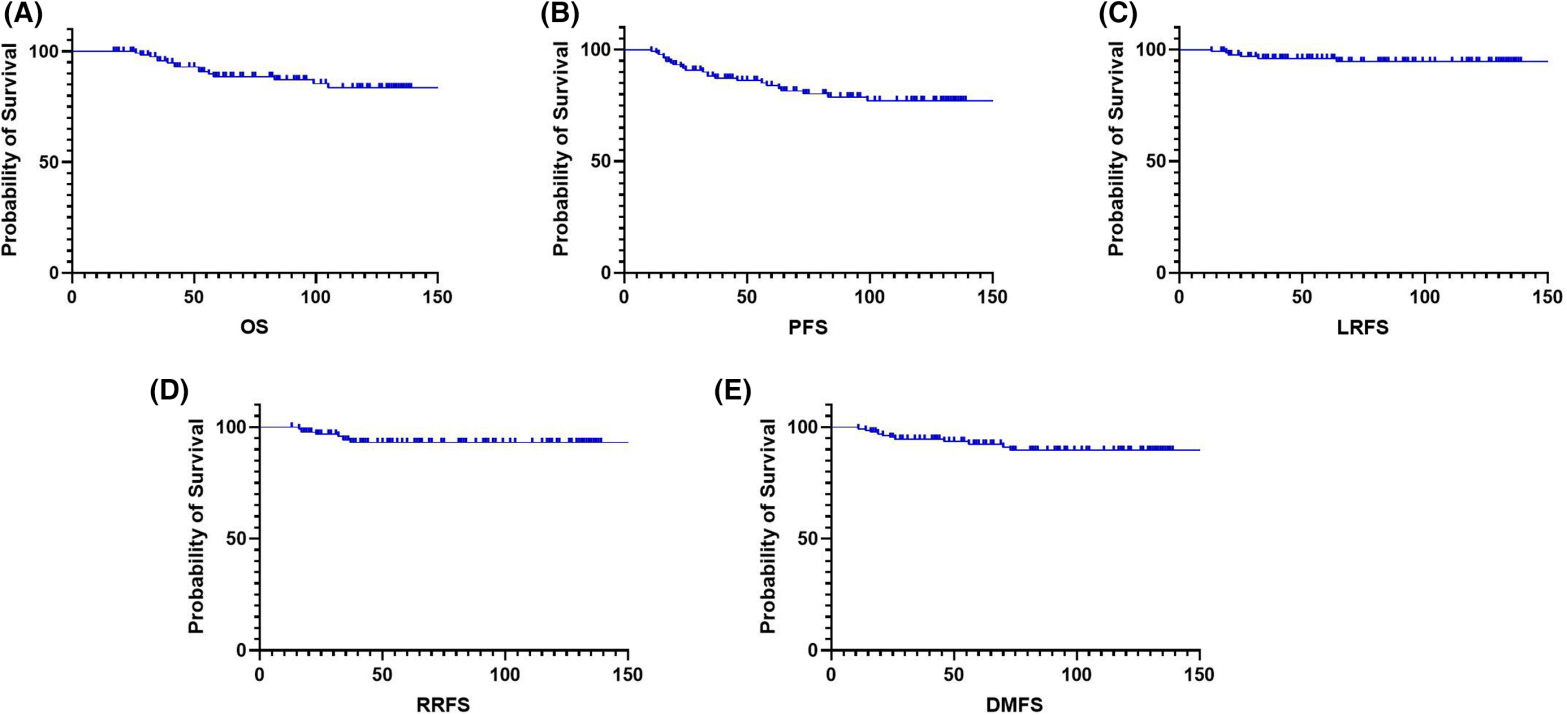
Kaplan–Meier survival curves of unilateral NPC patients. (A) Survival curves of overall survival (OS); (B) survival curves of progression‐free survival (PFS); (C) survival curves of local recurrence‐free survival (LRFS); (D) survival curves of regional recurrence‐free survival (RRFS); (E) survival curves of distant failure‐free survival (DMFS).

**FIGURE 3:**
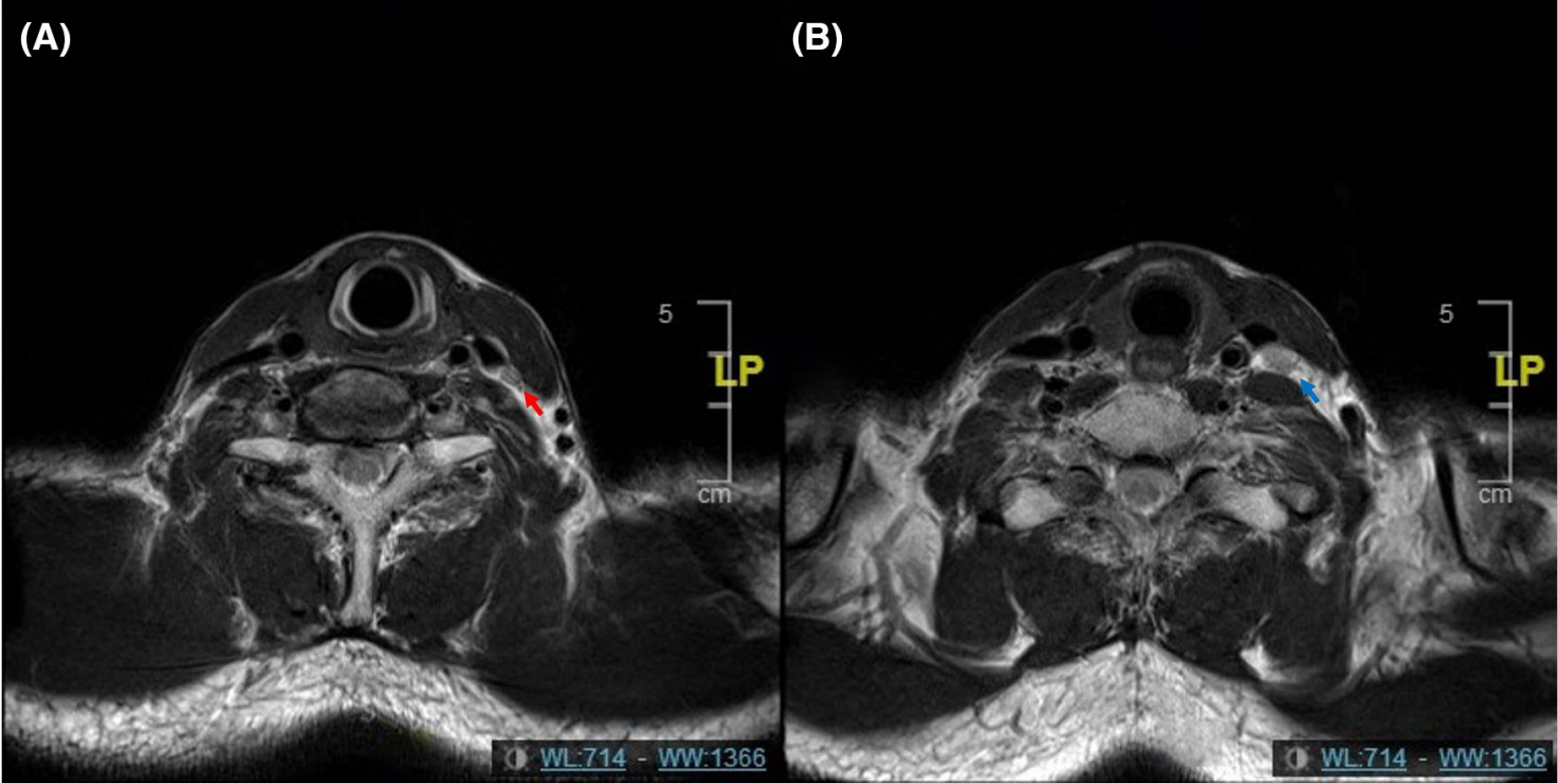
Regional recurrence illustration. The patient recurred in contralateral level IVa in the field of PCTV2. (A) Contralateral level IVa before treatment; (B) contralateral level IVa after recurrence. Red arrow: lymph node of contralateral level IVa before treatment; Blue arrow: lymph node of contralateral level IVa after recurrence.

Of 136 cases, four were lost to follow‐up; thus, 132 cases were included in the survival analysis, of which 16 cases died. Up to May 2022, the median follow‐up was 72 months (17–220 months). Figure 3 shows the 3‐, 5‐, and 10‐year overall survival (OS), progression‐free survival (PFS), local recurrence‐free survival (LRFS), regional recurrence‐free survival (RRFS), and distant failure‐free survival (DMFS). 3‐year OS, PFS, LRFS, RRFS, and DMFS were 95.7%, 88.1%, 96.0%, 94.1%, and 94.6% respectively; 5‐year OS, PFS, LRFS, RRFS, and DMFS were 88.6%, 83.8%, 96.0%, 93.1%, and 92.4% respectively; 10‐year OS, PFS, LRFS, RRFS, and DMFS were 83.6%, 77.1%, 94.6%, 93.1%, and 89.6%, respectively (Figure 2).

### 3.6 Failure patterns

A total of 21 cases had recurrent disease, including six cases of local recurrence, 8 cases of regional recurrence and 11 cases of distant metastasis (Table 5). Regional recurrence: six cases with in‐field failure relapsed in ipsilateral PGTVnd, of which, two cases also had out‐of‐field recurrence of ipsilateral parotid lymph nodes, one case recurred in contralateral level IVa which was in the field of PCTV2, and the other case relapsed in ipsilateral level Ib which was in the field of PCTVnd (Figure 3). Local recurrence: all local recurrent lesions were in‐field failures. Distant metastasis: a total of 11 cases, including 6 cases of single organ metastasis and 5 cases of multiple organ metastasis, these included 5 cases of bone metastasis, 5 cases of lung metastasis, 2 cases of liver metastasis, 1 case of parotid metastasis, 1 case of thyroid metastasis, and 1 case of abdominal para‐aortic lymph node metastasis.

**TABLE 5:** RLN failure analysis of recurrent patients.

	Stage	Primary site	Failure site	Local recurrence	Distant etastasis	Type of recurrence
Case 1	T3N2M0	R	Ipsilateral level Ib	No	No	PCTVnd in‐field
Case 2	T3N2M0	R	Ipsilateral level II &IV	No	No	PGTVnd in‐field PCTV2 in‐field
Case 3	T1N3M0	L	Ipsilateral level II & parotid LN	No	Parotid	PGTVnd in‐field & out‐of‐field
Case 4	T3N3M0	L	Ipsilateral level IV & V	No	No	PGTVnd in‐field
Case 5	T2N3M0	L	Ipsilateral level Vb	No	No	PGTVnd in‐field
Case 6	T2N2M0	R	Ipsilateral parotid LN	No	Bone & thyroid	Out‐of‐field
Case 7	T1N1M0	L	Ipsilateral level VIIa	No	No	PGTVnd in‐field
Case 8	T4N1M0	R	Ipsilateral level VIIa	Ipsilateral GTVnx	No	PGTVrpn in‐field

## 4 DISCUSSION

Tomita et al.^16^ reported a bilateral RLN metastasis rate of 18% in 76 patients with unilateral NPC on the patterns of RLN metastasis in newly diagnosed NPC patients. Wu et al.^18^ reported a bilateral RLN metastasis rate of 56.3% in 176 patients. In our study, the bilateral lymph node metastasis rate was 57.9%, indicating that unilateral NPC also carries a higher risk of bilateral RLN metastasis.

At present, there are few studies on RLN distribution of unilateral NPC. Tomita et al.^16^ found that the rate of bilateral RLN metastasis in 56 patients with unilateral NPC was 18%. Li et al.^17^ confirmed that about 37.5% had bilateral RLN metastasis. Wu et al.^18^ reported that the rate of bilateral RLN metastasis in unilateral NPC was 53.4%. Similarly, the rate was 57.9% in our study, suggesting that unilateral NPC also has a higher risk of bilateral RLN metastasis. Sun et al.^19^ reported that bilateral RLN metastasis in 112 cases of unilateral NPC accounted for only 8%, which may be related to the lower proportion with locally advanced stage. However, the total RLN metastasis rate was only 54%, which was lower than 80% reported in unilateral NPC studies^1^, ^10^ and 92.6% in our study. However, which RLN on the contralateral side of the neck require prophylactic irradiation is still uncertain.

Previous literature^20^, ^21^, ^22^, ^23^ reported that RLN metastasis from the upper neck to the lower neck metastasis rate gradually decreased, rare RLN skipping metastasis in NPC patients. Our study showed that the ipsilateral and contralateral RLNs of unilateral NPC also followed the above rule of metastasis. Previous study^24^ found metastases usually spread down from higher level RLN to lower in the ipsilateral side rather than contralateral or bilateral metastases. Similarly, the rate of RLN metastasis in all regions of the contralateral side was lower than that of the ipsilateral side in our study. Furthermore, it was found that the risk of RLN metastasis in contralateral level VIIa and II was highest, reaching 41.9% and 38.2%, respectively. Further analysis of the correlation between contralateral and ipsilateral RLN metastasis showed that there was a significant correlation between contralateral level VIIa and ipsilateral level VIIa metastasis, contralateral level II and ipsilateral level II and III metastasis (all the *p* < 0.05). Contralateral level III also had a moderate risk of RLN metastasis, with an incidence of approximately 8.1%. Therefore, it is recommended that unilateral NPC should be given preventive irradiation in contralateral level VIIa, II and III.

In this study, the rate of RLN metastasis in the contralateral level IVa was lower (0.7%), and similar results were observed in previous studies^16^, ^18^, ^19^ in unilateral NPC. Numerous studies^11^, ^24^, ^25^ on selective neck prophylactic irradiation of NPC have shown remarkable nodal control that nodal relapse on the contralateral level IVa were extremely low (0%–0.7%). Consistently, our result found that only one patient developed recurrence in contralateral level IVa. Based on the above conclusions, the LNs in the contralateral level IVa in unilateral NPC are low risk areas (<5%), and contralateral level IVa‐sparing IMRT is feasible with unilateral cervical LN metastasis.

Compared with other head and neck squamous cell carcinomas, patients with NPC are more likely to have metastasis in level Va (11.1%–25%),^20^, ^26^, ^27^, ^28^ but the rate of contralateral and ipsilateral level Va metastasis has not been further investigated. Although level Va is a moderate risk region, it is still lower than that in the upper jugular level II–III. The law of level Va transfer and its relationship with other regions are not clear. According to an anatomical study,^29^, ^30^ level Va may mainly receive the capillary network from the Eustachian tonsil (the lateral wall of the nasopharynx), while the jugular chain contains the capillary network from the Eustachian tonsil and the pharyngeal tonsil (the lateral and the posterior wall of the nasopharynx), so the metastasis rate in level V is lower than that of jugular chain LNs. In this study, no contralateral level Va metastasis was found, which was consistent with the results of N Tomita et al.^16^ Sun et al.^19^ summarized the distribution of RLN metastasis in 112 patients with unilateral NPC and found that contralateral level Va metastasis occurred in only 1 case (<1%). This indicates that contralateral level Va metastasis is rare in patients with unilateral NPC. Therefore, contralateral level Va may not need routine prophylactic irradiation. Whereas, limited to the sample size in the present study, there are still doubts that the metastasis of contralateral level Va regarding bilateral extensive LN metastasis. So far, there are few reports on the metastasis of level Va in the case of extensive bilateral RLN metastasis of unilateral NPC. Previous studies^31^, ^32^ on LN metastasis of hypopharyngeal carcinoma showed that LN metastasis in level V was rare (5%), and it often occurred with extensive ipsilateral LN metastasis. Our results showed the same relationship between contralateral level V and other level LNs. Patients with ipsilateral level V metastasis (11.0%) were all staged N2‐3, and ipsilateral level V metastasis was also accompanied by metastasis at other levels to varying degrees (100% with ipsilateral level VIIa metastasis, 80% with level II metastasis, 80% with level III metastasis). With regard to the contralateral site, the metastasis rate of level III was only 8.1%, and that of level IVa was 0.7%. Whether metastasis of contralateral level Va may also occur in the case of extensive metastasis of other levels remains to be further investigated in the future.

Retropharyngeal LNs are one of the first levels of metastases from NPC. Previous studies on 3100 NPC cases^33^ showed only six (0.2%) patients with medial group retropharyngeal LN involvement. The results of another study^28^were also consistent with these findings. At present, most radiation oncologists^6^ support excluding the medial group from CTV2. Our results also confirmed this with no recurrence in the bilateral medial group without prophylactic irradiation.

The 5‐year RRFS was 93.1% in the present study, which is similar to that in previous studies (91%–92.5%).^34^, ^35^ Eight cases of RLN recurrence, of which one patient had ipsilateral level II metastatic PGTVnd in‐field recurrence, along with a contralateral level IV suspected LN recurrence (Table 5). The patient was staged as T3N2M0, with ipsilateral level VIIa, IVa, and contralateral level II–III metastasis. The suspected LN was located in contralateral level IV, with a maximum short diameter of 3 mm, delineated in the range of CTV2, and prescribed 45Gy (1.8Gy/F). Another patient with in‐field recurrence of ipsilateral level Ib LN was prescribed 64Gy (2.0Gy/F). The clinical stage was T3N2M0 III stage, ipsilateral level VIIa and level II–III, and contralateral VIIa and level II metastasis. Before treatment, MRI showed a suspected enlarged LN in ipsilateral level Ib, with a maximum short diameter of 5 mm. The NCCN guidelines recommend prescription doses ranging from 44–50Gy/2.0Gy/F to 54–63Gy/1.6–1.8Gy/F.^36^ However, it is still unclear whether the radiation dose should be given to clinically detected suspected LNs. Kuong et al.^37^ reported an NPC patient with suspected LN metastasis of diameter < 10 mm before treatment, which still recurred 27 months after 64Gy irradiation. For head and neck tumors, postoperative radiotherapy of 66Gy is prescribed for highly suspicious residual disease, suggesting that a higher dose of prophylactic irradiation may be needed for highly suspected LNs. Therefore, in recent years, we have tried to prescribe 63Gy‐65Gy/31‐32F for high‐risk suspected metastatic LN as CTVnd.

Several limitations should be taken into consideration. First, late toxicity and the cause of death are not detailed enough over the long‐term. Therefore, we failed to confirm the relevant evaluation and analysis. Second, this is a retrospective study with a small sample size. The results of the study need to be verified by further prospective clinical studies.

## 5 CONCLUSION

In each RLN region, the metastasis rate of contralateral RLNs was lower than that of the ipsilateral rate for unilateral NPC. The metastasis of RLNs for unilateral NPC were from the upper neck to the lower neck. Metastasis in contralateral lower neck RLNs (level IVa, IVb, Vb, and Vc) were at low‐risk areas, and level Va was rare. Therefore, upper neck RLNs needed to be included in CTV, while reduced prophylactic irradiation in contralateral lower neck LNs and contralateral level Va could be recommended for N0‐1 patients.

## AUTHOR CONTRIBUTIONS

**Feifei Lin:** Conceptualization (equal); data curation (equal); formal analysis (lead); investigation (equal); methodology (equal); project administration (equal); resources (equal); software (lead); supervision (equal); validation (equal); visualization (lead); writing – original draft (lead); writing – review and editing (equal). **Zichen Qiu:** Data curation (equal); formal analysis (equal); investigation (equal); methodology (equal); resources (equal); software (equal); validation (equal); visualization (equal); writing – original draft (equal). **Dehuan Xie:** Conceptualization (equal); data curation (equal); investigation (equal); resources (equal); software (equal); validation (equal); writing – original draft (equal). **Xiong Zhou:** Data curation (equal); investigation (equal); methodology (equal); software (equal); validation (equal); visualization (equal). **Lei Wang:** Formal analysis (equal); investigation (equal); software (equal); validation (equal). **Zheng Wu:** Investigation (equal); project administration (equal); software (equal); writing – review and editing (equal). **Wanqin Cheng:** Investigation (equal); methodology (equal); resources (equal); software (equal). **Shaowen Lyu:** Methodology (equal); resources (equal); software (equal). **Yong Su:** Conceptualization (lead); data curation (equal); formal analysis (lead); investigation (equal); methodology (equal); project administration (lead); resources (equal); supervision (lead); validation (equal); visualization (equal); writing – review and editing (lead). **Yalan Tao:** Conceptualization (lead); data curation (equal); formal analysis (lead); investigation (equal); methodology (equal); project administration (lead); resources (equal); supervision (equal); validation (lead); visualization (equal); writing – original draft (equal).

## FUNDING INFORMATION

The authors declare that they had no funding for this study.

## CONFLICT OF INTEREST STATEMENT

The authors declare that they have no competing interests.

## ETHICAL STATEMENT AND CONSENT TO PARTICIPATE

Retrospective ethical approval was obtained from the institutional ethics committee of the Sun Yat‐sen University Cancer Center, with the approval number YB2019‐169‐01. Written informed treatment consents were required from all patients with respect to chemotherapy and/or radiotherapy.

## Data Availability

The datasets used and/or analyzed during the current study were uploaded onto the Research Data Deposit public platform (www. researchdata. org. cn) with approval number of RDDA 2210090001.
